# Supplementary figures and images for: Decoding the neural dynamics of free choice in humans
Source: PLoS Biol. 2020 Dec 10;18(12):e3000864. doi: 10.1371/journal.pbio.3000864 (PMC7755286; doi:10.1371/journal.pbio.3000864)

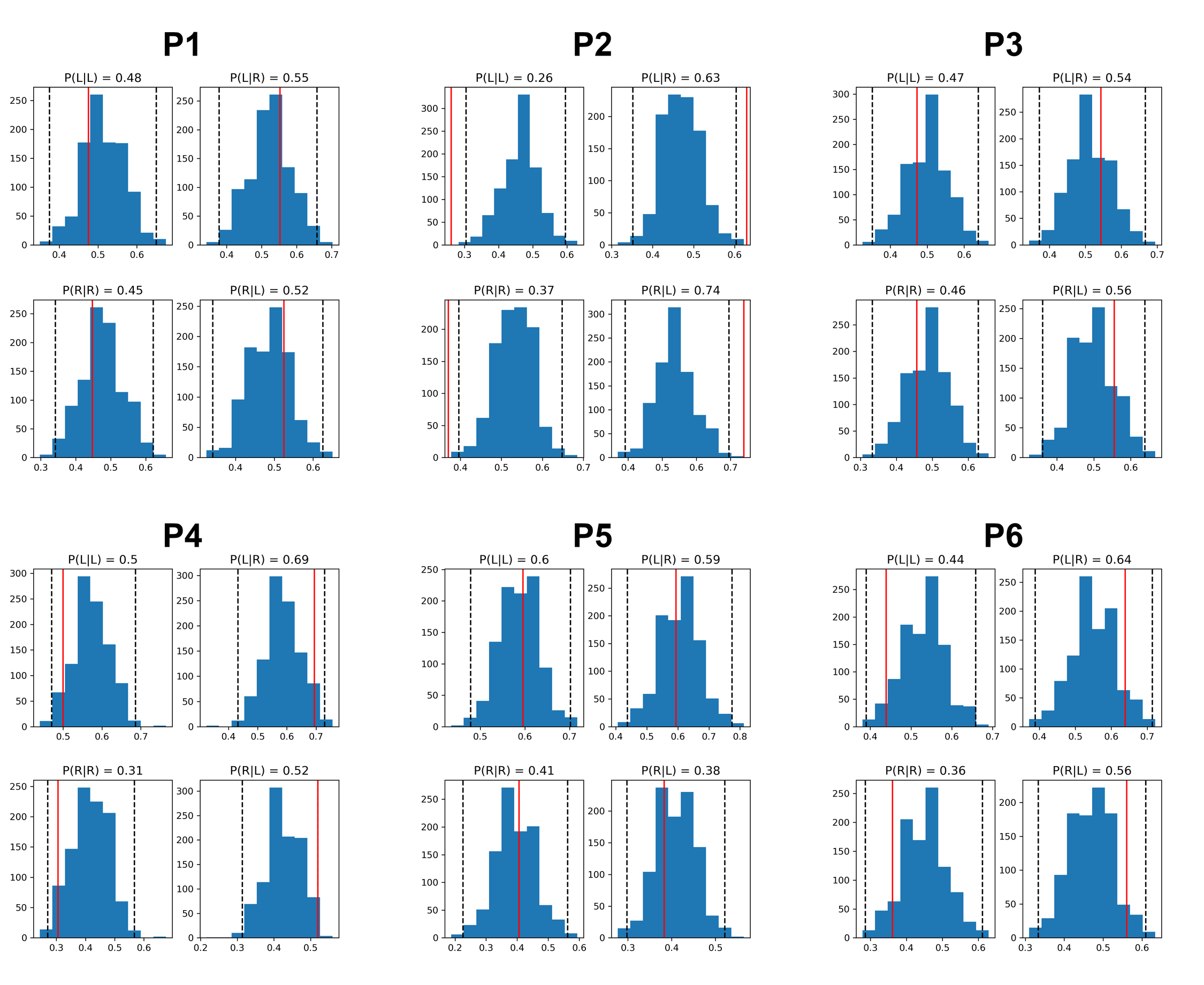

Supplement: S1 Fig — Conditional probabilities (n-1) were computed to determine whether the saccade direction (Left, L or Right, R) of trial n-1 influenced trial n in the Free-choice condition. For each participant, the value of conditional probabilities P(L|L), P(L|R), P(R|R) and P(R|L) are shown. The statistical significance of the obtained conditional probabilities was evaluated by computing statistical thresholds using permutation tests (n = 1,000, p < 0.001). In other words, a null distribution is generated by repeatedly (n = 1,000) computing conditional probabilities for each participant obtained after randomly permuting class labels (Left and Right). We show that P2 was the only participant demonstrating a significant alternating behavior between left and right choices during the Free condition. (TIF) [file pbio.3000864.s007.tif]

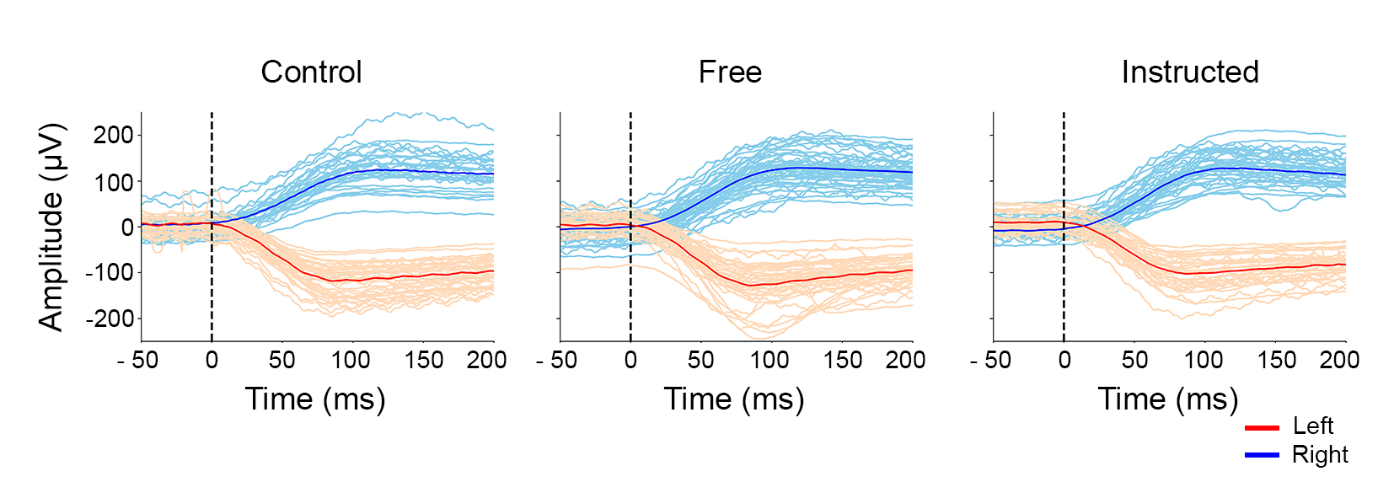

Supplement: S2 Fig — Thin lines represent EOG traces for all trials and thick lines represent mean EOG traces locked on saccade onset for each condition (Control, Free, Instructed). EOG, electrooculogram. (TIF) [file pbio.3000864.s008.tif]

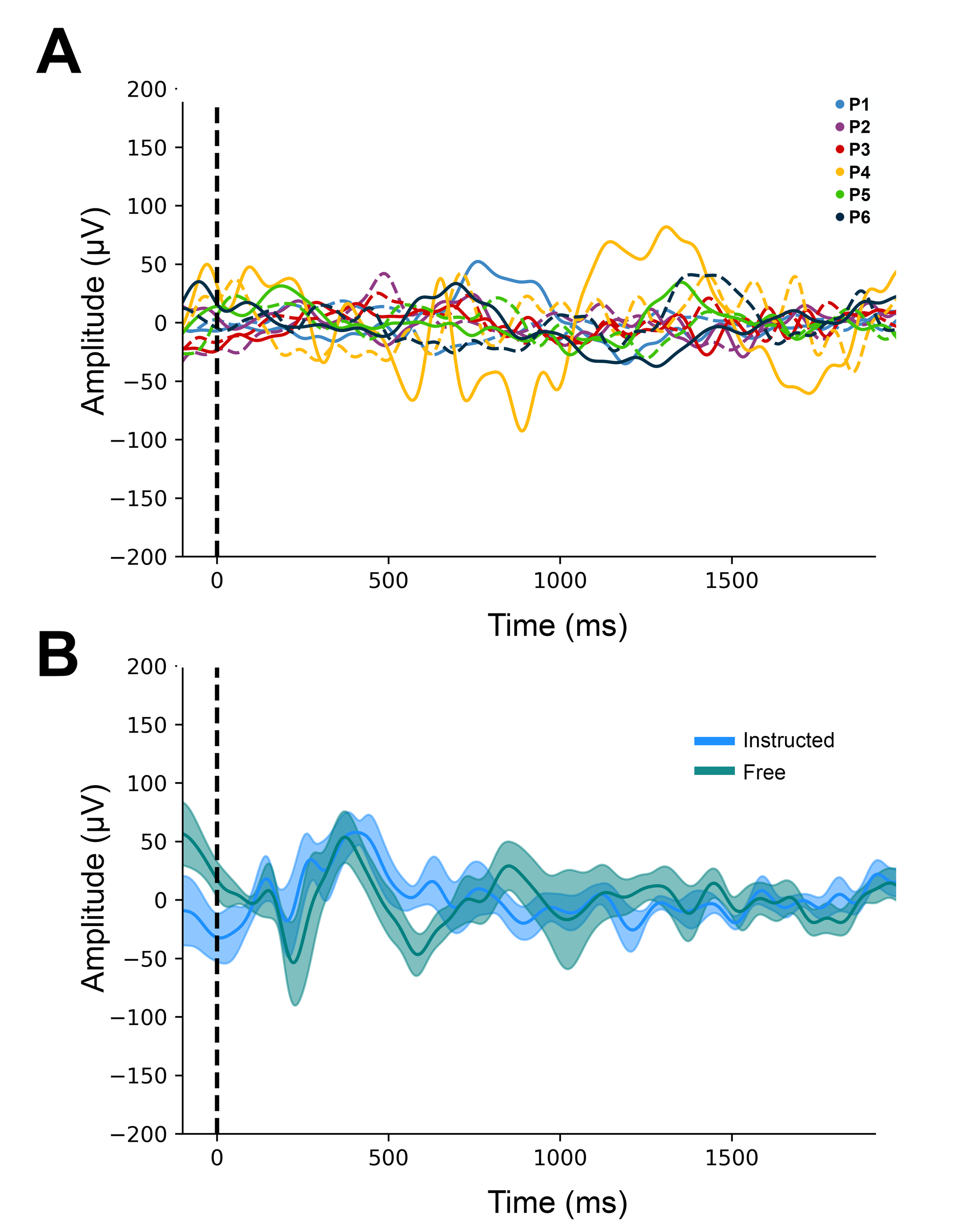

Supplement: S3 Fig — (A) Mean EOG traces for all participants locked t stimulus onset (Cue 1) during the delay period. Each color is associated with a participant, dashed lines represent left saccades and full lines represent right saccades for all conditions. (B) Mean EOG traces for Instructed and Free conditions during the delay period. The data underlying panel B can be found in S1 Data. (TIF) [file pbio.3000864.s009.tif]

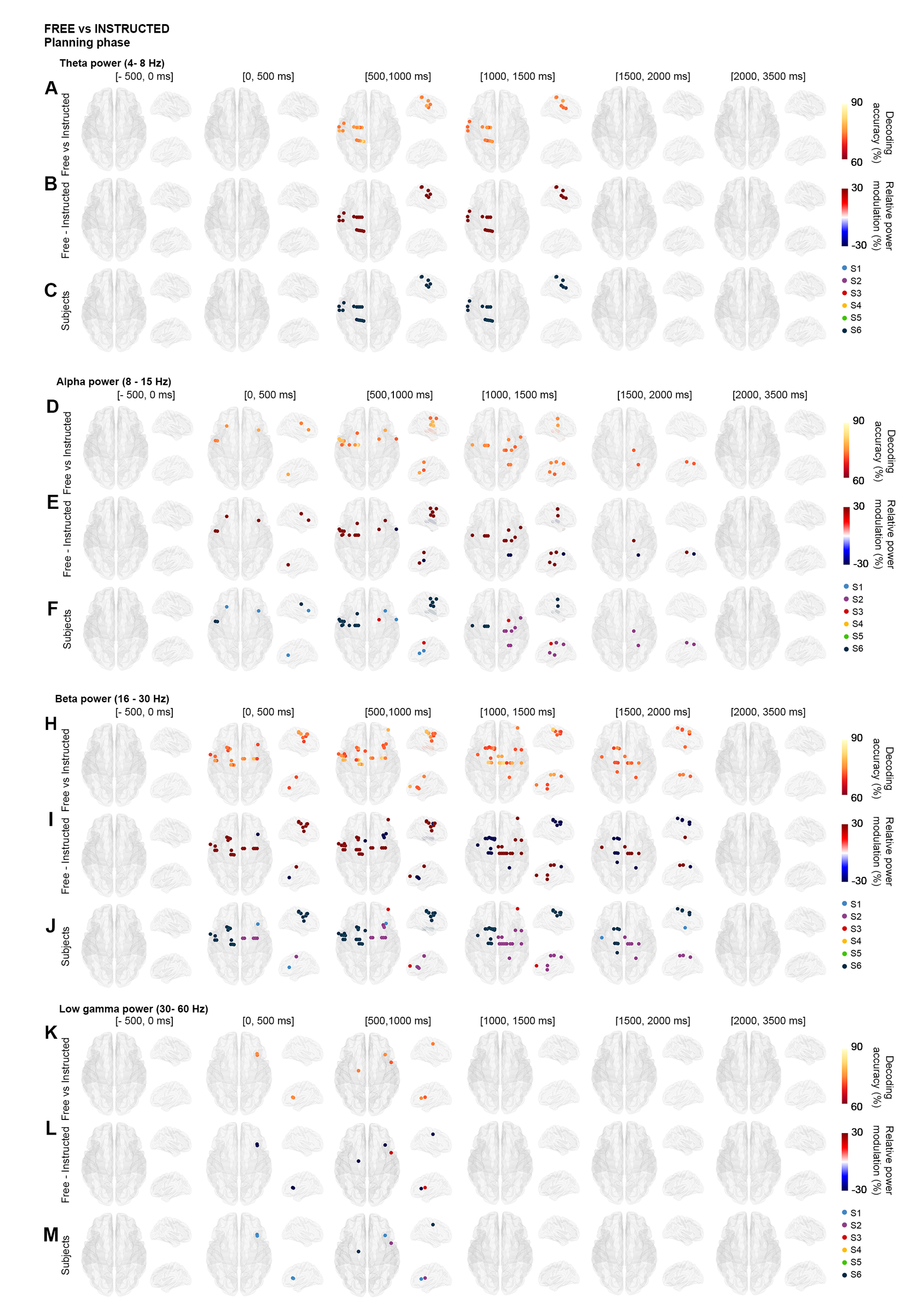

Supplement: S4 Fig — We compared high-frequency neuronal activity in θ (4–8 Hz), α (8–15 Hz), β (16–30 Hz), and low-γ (30–60 Hz) frequency bands for the Free and Instructed conditions during the planning phase in different time windows: baseline = −500 to 0 milliseconds; 0 to 500 milliseconds; 500 to 1,000 milliseconds; 1,000 to 1,500 milliseconds; 1,500 to 2,000 milliseconds; and 2,000 to 3,500 milliseconds (A, D, H, K). Electrode-specific significant decoding accuracies (corrected across electrodes, time, and frequency bands using exhaustive permutations corrected with maximum statistics at p < 0.01), (B, E, I, L) relative power changes (relative change = [Free −Instructed]/Instructed), and (C, F, J, M, P) colors belonging to individual participants are mapped to the corresponding electrode positions on transparent 3D brain images. (TIF) [file pbio.3000864.s010.tif]

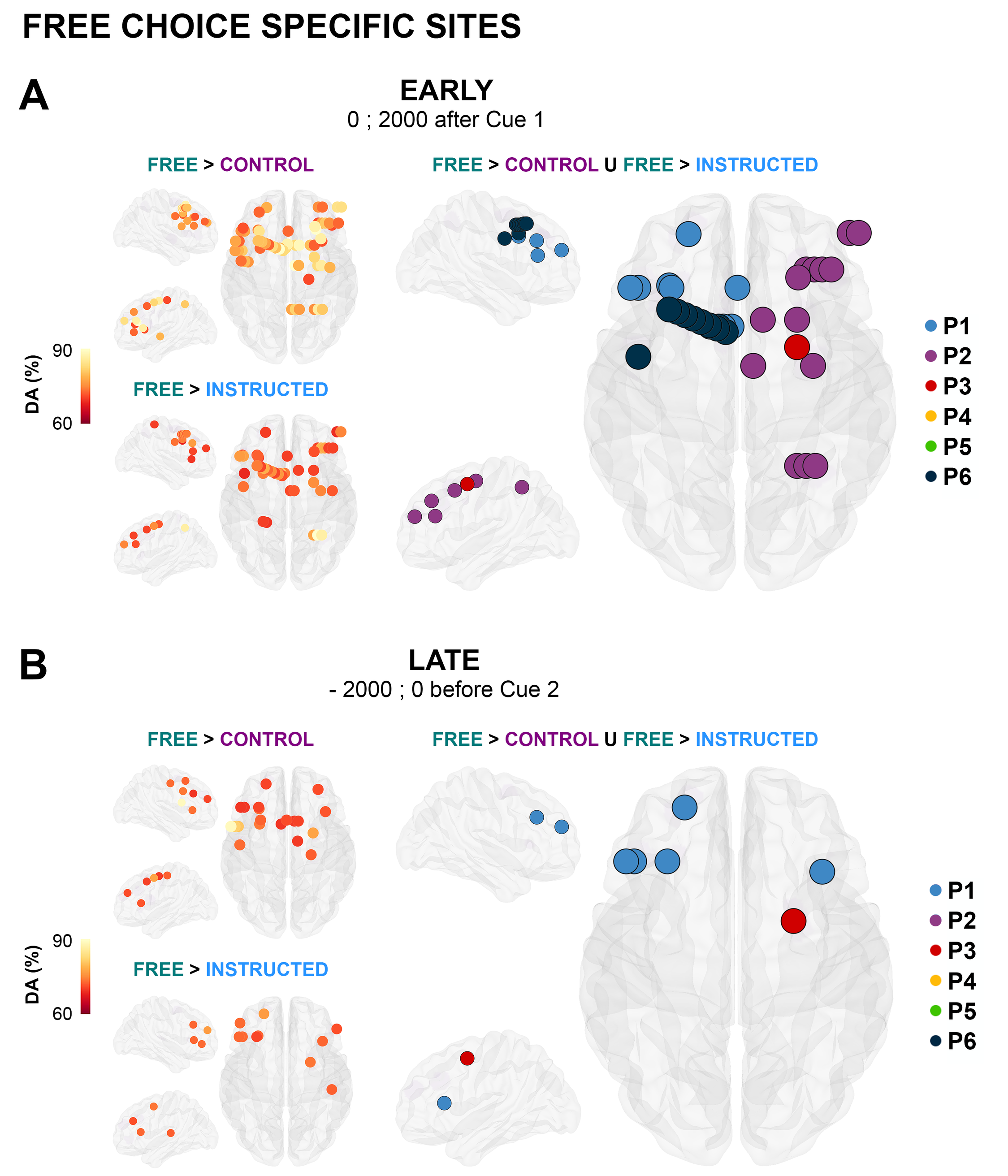

Supplement: S5 Fig — In A, electrodes with significant decoding accuracies (p < 0.01, corrected with permutations using maximum statistics across electrodes, frequency bands and time) for all participants are mapped on transparent 3D brain images when HG activity is significantly stronger in the Free condition than in the Control condition (first row), and when HG activity is significantly stronger in the Free condition than in the Instructed condition (second row) during the delay period, from 0 to 2,000 milliseconds after Cue 1 (i.e., early). We isolated a network of regions specifically involved in Free decisions showed in the right panel using a conjunction analysis (Free > Control U Free > Instructed). Electrodes are colored based on the participant to which they belong. B. The same analysis was conducted for the late part of the delay period, from −2,000 to 0 milliseconds second before Cue 2. (TIF) [file pbio.3000864.s011.tif]

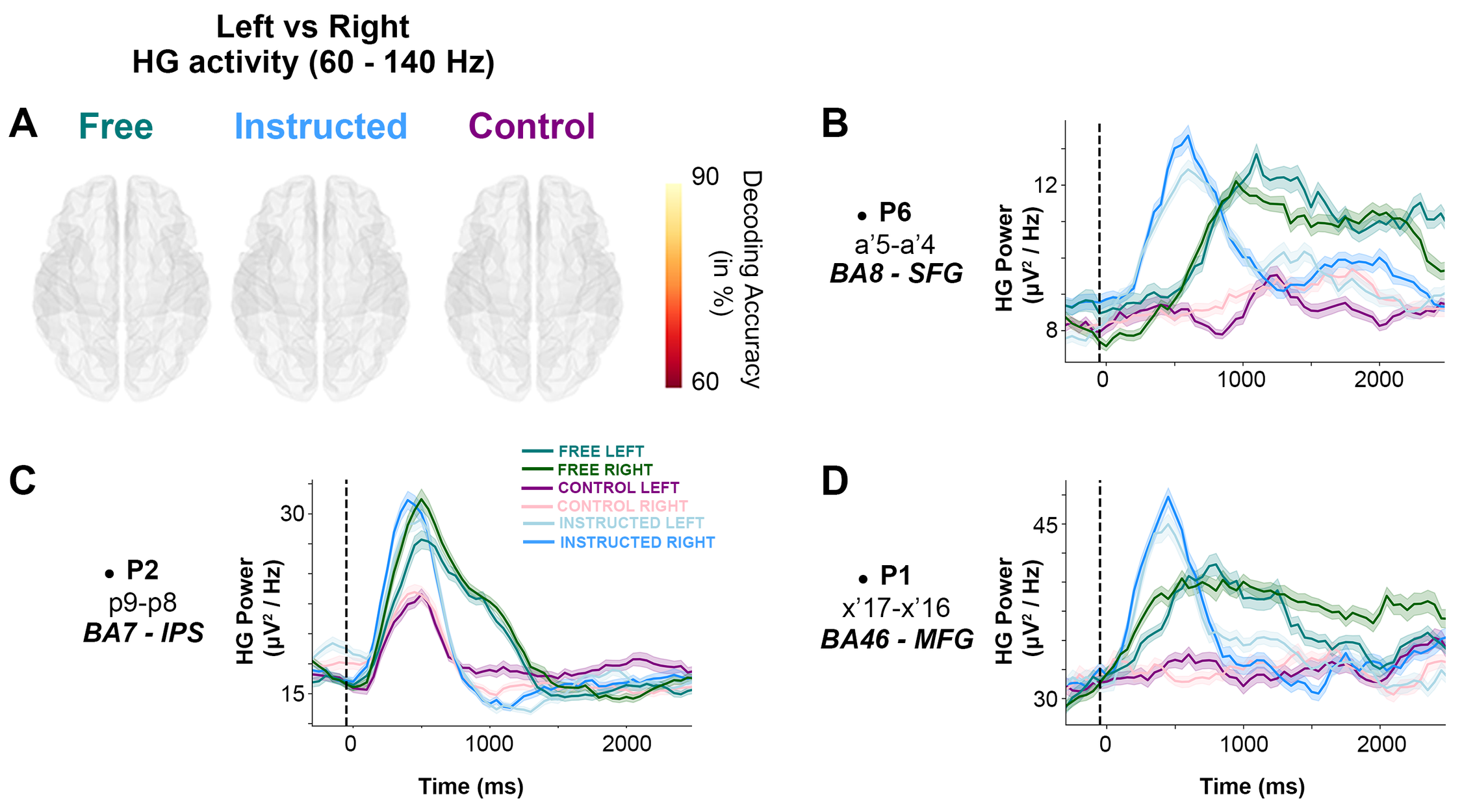

Supplement: S6 Fig — A. Electrodes with significant decoding (corrected across electrodes, time and frequency bands using exhaustive permutations corrected with maximum statistics at p < 0.01) when comparing HG activity between left and right choices in the Free, Instructed, and Control conditions during the delay period phase (0 to 2,000 milliseconds after Cue 1)) for all participants and mapped on transparent 3D brain images. B, C, D. For 3 individual electrodes, we plotted HG activity over time for Free (left and right), Instructed (left and right) and Control (left and right) conditions. We show that the capacity of electrodes to successfully decode Free versus Control and Free versus Instructed conditions based on HG activity on a single-trial basis is not determined by the content (left or right) of decisions. The data underlying panels B, C, and D can be found in S1 Data. HG, high-gamma. (TIF) [file pbio.3000864.s012.tif]

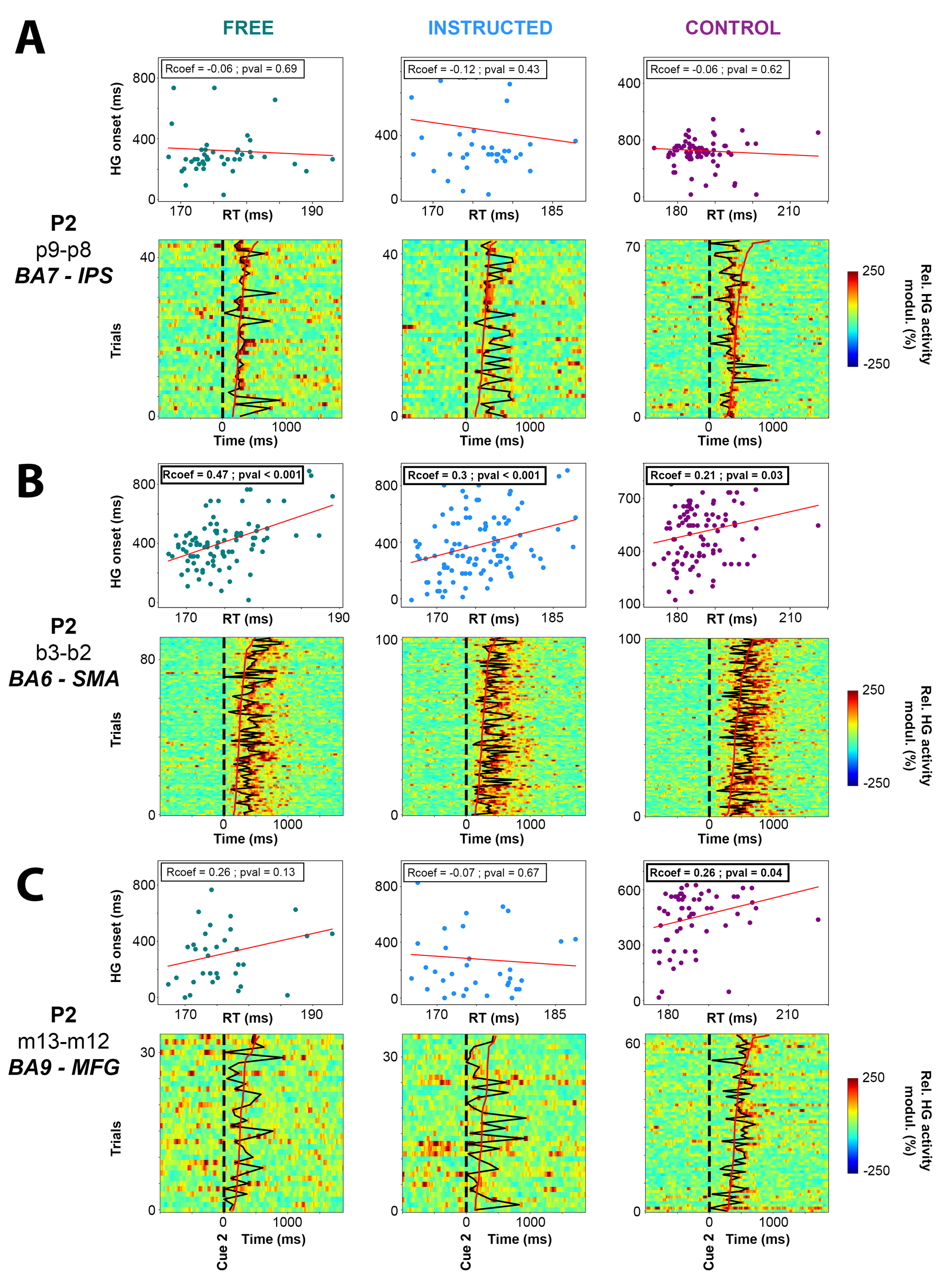

Supplement: S7 Fig — For 3 example electrodes located in the IPS (A), SMA (B), and MFG (C): correlations between reaction times and the latencies of HG activity onset (upper rows) and single-trial plots (lower rows) are show for the Free, Instructed, and Control conditions. On single-trial plots, trials are sorted with respect to RTs. RT latencies are represented by continuous red lines, and the latencies of HG activity onset are represented by continuous black lines (see Material and methods). The data underlying this Figure can be found in S1 Data. HG, high-gamma; IPS, intraparietal sulcus; MFG,; RT, reaction time; SMA, supplementary motor area. (TIF) [file pbio.3000864.s013.tif]
